# Surveillance of Seasonal Influenza Viruses in Kazakhstan (2020–2025)

**DOI:** 10.3390/v18040441

**Published:** 2026-04-07

**Authors:** Tatyana Glebova, Nailya Klivleyeva, Nuray Ongarbayeva, Assem Baimukhametova, Nurbol Saktaganov, Mereke Kalkozhayeva, Kobey Karamendin, Indira Ibragimova, Madisha Sagatova, Aknur Mutaliyeva, Altynay Gabiden, Richard Webby

**Affiliations:** 1 The Research and Production Center for Microbiology and Virology, Almaty 050010, Kazakhstan; nuray.syrlybay@gmail.com (N.O.);; 2 Kazakhstan-Russian Medical University, Postgraduate Cardiology Course, Almaty 050000, Kazakhstan; 3 The East Kazakhstan Regional Branch of National Center for Expertise, Ust-Kamenogorsk 070003, Kazakhstan; 4 National Center of Public Health Care, The Ministry of Health of the Republic of Kazakhstan, Almaty 050008, Kazakhstan; mutaliyeva.a.s@gmail.com (A.M.);; 5 Department of Infectious Diseases, St. Jude Children’s Research Hospital, Memphis, TN 38105-3678, USA

**Keywords:** respiratory infection, influenza, virus, antigen, antibody

## Abstract

Influenza viruses are significant causes of acute respiratory infections, often leading to severe health issues and mortality. These viruses undergo continuous mutations and genetic reassortments, resulting in annual epidemics and potential pandemics. The A(H3N2) strains exhibit high genetic and antigenic variability, that influence vaccine efficacy. This study aimed to assess the prevalence of influenza viruses, including A(H3N2) strains, in Kazakhstan during 2020–2025. The study used nasopharyngeal swab and serum samples obtained from patients. The presence of influenza virus antigens in nasopharyngeal swabs was analyzed using real-time polymerase chain reaction. The level of specific antibodies in the blood serum was determined using the hemagglutination inhibition reaction and enzyme-linked immunosorbent assay methods. Influenza A/H1N1, A/H3N2 and B viruses were diagnosed using real-time PCR. Antibodies to A/H1N1pdm09, A/H3N2 and B were detected in serological studies. Our studies revealed a trend toward seasonal patterns in influenza A viruses circulation. Therefore, it was established that the A/H3N2 strains dominated in Kazakhstan during the 2021–2022 and 2023–2024 epidemic seasons. The 2023–2024 strains belong to the specific genetic clade J.2 or 3C.2a1b.2a.2a.3a.1. These studies confirmed the role of influenza viruses in the etiology of respiratory infections and emphasized the need to continue monitoring their spread in Kazakhstan.

## 1. Introduction

Acute respiratory infections pose a serious threat to public health worldwide and are associated with both viral and bacterial pathogens [1,2]. Viral pathogens of acute respiratory infections are one of the main causes of hospitalization in children. Complications can reveal as croup, bronchiolitis, pneumonia, exacerbation of asthma, and even lead to more serious consequences, including respiratory failure and acute respiratory distress syndrome [3,4]. The most important pathogens are influenza A and B viruses, parainfluenza, respiratory syncytial virus, adenoviruses, rhinoviruses and coronaviruses, including COVID-19 [5,6,7]. Influenza viruses circulate continuously among the population, causing seasonal increases in incidence and periodically becoming epidemics and even pandemics, and are associated with some of the highest mortality rates in children and at-risk individuals [3,4,6,8].

Furthermore, influenza A viruses (IAVs) and coronaviruses have caused at least five pandemics in the global history of fatal infections since the early 20th century. Four of these were caused by the introduction of new influenza viruses into the human population: H1N1 in 1918 and 2009, H2N2 in 1957, and H3N2 in 1968. Following each pandemic, influenza viruses transitioned to a seasonal form with an annual increase in respiratory illness worldwide, resulting in 3–5 million severe cases and 290,000 to 650,000 deaths [9,10].

The H3N2 IAV, which caused the third influenza pandemic of the last century, emerged in Hong Kong in 1968 (A/Hong Kong/1/1968) and is associated with over a million deaths worldwide. The emergence of this viral strain is believed to have occurred as a result of reassortment events between previously circulating human H2N2 viruses and avian H3N2 viruses. The resulting reassortant acquired the ability to infect and transmit from human to human. The origin of the virus from avian H2N2 and H3N2 strains was confirmed by whole-genome analysis of hemagglutinin (HA), neuraminidase (NA) and PB1 fragments. Subsequently, by 1971, H3N2 viruses had completely displaced H2N2 strains from the human population and were involved in seasonal circulation, causing numerous epidemics with high morbidity and mortality [11].

A(H3N2) strains are characterized by high genetic and antigenic variability, leading to frequent changes in the composition of their surface proteins (HA and NA), which impacts vaccine effectiveness. Currently, these viruses often circulate together with influenza A(H1N1) and influenza B viruses and can cause epidemics. The A(H3N2) virus is associated with an increased risk of hospitalization and mortality, with children and the elderly at greatest risk, as well as nosocomial spread [12].

Since its initial emergence in 1968, the influenza A(H3N2) virus has undergone significant evolution. Numerous severe outbreaks, low vaccination efficacy, and recurring seasonal fluctuations in vaccine strains have been associated with changes in A(H3N2) viruses. From October 2021 to February 2022, influenza A(H3N2) was the most prevalent virus in Kazakhstan [13]. Subclade 3C.2a1b.2a.2 has been shown to be widespread worldwide and has now diverged into several subclades [14]. This study assessed the prevalence of influenza viruses and molecular analysis of modern H3N2 IAV strains during five consecutive epidemic seasons.

## 2. Materials and Methods

### 2.1. Clinical Samples Collection

During the 2019–2025 epidemic seasons, 2200 nasopharyngeal swabs and 1465 blood serum samples were collected from infected individuals at healthcare facilities located in various regions of Kazakhstan. Biological samples were collected by medical workers from patients with symptoms such as fever, cough, sore throat, runny nose, muscle or body aches, headache, or fatigue.

The study was conducted in accordance with the Declaration of Helsinki and was approved by the Research Ethics Committee of the Scientific and Production Center for Microbiology and Virology, Almaty, Kazakhstan (Protocol No. 19 of the local bioethics committee meeting dated 1 April 2025). All nasopharyngeal swab samples that tested positive for IAV by real-time polymerase chain reaction (rtRT-PCR) were processed in accordance with ethical standards, ensuring patient confidentiality.

Sample collection was carried out in accordance with WHO recommendations [15]. Each sample was mixed with 2 mL of virus transport medium (minimal essential medium (Gibco, Invitrogen, Grand Island, NY, USA) and transported refrigerated. Samples were vortexed for 15 s, centrifuged at 1000× *g* for 10 min at 4 °C, aliquoted, and stored at −80 °C until further analysis. Serum was separated by centrifugation at 400 *g* for 15 min and stored at −20 °C until analysis.

### 2.2. Typing, Detection, and Sequencing of Influenza a Virus

#### 2.2.1. Antigen Detection and Typing

Viral RNA was extracted using the QIAamp Viral RNA Mini Kit (Qiagen, Hilden, Germany) according to the manufacturer’s instructions. Samples were tested for influenza viruses using specific real-time PCR assays on a Rotor-Gene Q6 plex (QIAGEN, Hilden, Germany) according to Centers for Disease Control and Prevention (CDC) protocols. In cases where the influenza virus type was identified, complete HA and NA gene sequencing and corresponding phylogenetic analysis were performed.

#### 2.2.2. Antibody Detection in Serological Studies

To remove nonspecific inhibitors, a receptor-degrading enzyme was added to the serum samples at a working dilution of 1:50 in a 3:1 ratio (enzyme/serum) and incubated for 18 h at 37 °C. For testing, the serum was diluted with phosphate-buffered saline and heated for 30 min at 56 °C. The presence of specific antibodies to influenza virus HA in the serum was determined using the hemagglutination inhibition test (HI) and enzyme-linked immunosorbent assay (ELISA) in accordance with WHO recommendations [15,16]. The HI reaction was performed using commercial diagnostic kits: A/Michigan/45/2015 (H1N1)pdm, A/Singapore/INFIMH-16-0019/2016 (H3N2), B/Phuket/3073/13 and B/Colorado/06/2017 (Federal State Budgetary Institution “Research Institute of Influenza” of the Ministry of Health and Social Development of the Russian Federation, St. Petersburg, Russia). ELISA was performed using kits for the detection of antibodies to influenza viruses A/H1N1, A/H3N2 and influenza B viruses from the same manufacturer.

#### 2.2.3. Amplification of Full-Length HA and NA Genes

Whole influenza virus genome amplification was performed by one-step reverse transcription and polymerase chain reaction using the Biomaster RT-PCR-RV (2×) RT-PCR reagent kit (Biolabmix, Novosibirsk, Russia) in accordance with modified protocols for influenza viruses [17,18] on Bio-Rad CFX96 Touch systems (Hercules, CA, USA). The resulting PCR products (fragments) from whole-genome influenza virus amplification were verified by electrophoresis in 2% agarose gel in TAE buffer.

Library preparation for whole-genome sequencing was performed using the Illumina DNA Prep kit (Illumina, San Diego, CA, USA) according to the manufacturer’s recommendations. Samples were indexed using the Illumina IDT^®^ Illumina^®^ DNA/RNA UD Indexes Set A/B/C/D (Illumina, San Diego, CA, USA). Whole-genome amplification products of the influenza virus were purified using AMPure XP magnetic beads (Beckman, Indianapolis, IN, USA). The concentration of double-stranded DNA in the PCR products was determined using a Qubit fluorimeter and the Qubit HS dsDNA kit (Life Technologies, Carlsbad, CA, USA). Purified cDNA was diluted to a concentration of 0.2 ng/μL and then used for library preparation using an Illumina reagent kit (San Diego, CA, USA). Sequencing was performed using the Illumina NextSeq P2 100-cycle kit (Illumina, San Diego, CA, USA) on an Illumina NextSeq 2000 sequencer (Illumina, San Diego, CA, USA).

### 2.3. Sequence and Phylogenetic Analysis

Multiple alignment of the complete HA and NA genes was performed using the ClustalW (version 2.1) algorithm implemented in Mega (version 12). The analysis compared A/H3N2 strains with international reference and vaccine strains obtained from the GISAID (02/09/2025) and GenBank databases (25/08/2025). Sequence variations, including amino acid substitutions, insertions/deletions, nucleotide divergences, and potential N- and O-glycosylation sites, were identified using Nextclade v3.18.1 [19]. Phylogenetic trees were constructed using MEGA v11 (Pennsylvania State University, University Park, PA, USA) with 1000 bootstrap replicates to assess branch robustness [20]. The HA gene phylogeny was inferred using the maximum likelihood method and the Hasegawa–Kishino–Yano (1985) model [21]. The NA gene phylogeny was inferred using the Tamura (1992) model [22]. The percentage of replication trees in which related taxa clustered together (1000 replications) are shown next to branches [23]. Differences in evolutionary rates between sites were modeled using a 5-category discrete gamma distribution (+G, parameter = 0.3416) for HA and (+G, parameter = 0.7384) for NA. Evolutionary analyses were performed in MEGA12(version 12.1 [24] using up to 4 parallel computing threads.

### 2.4. Statistical Analysis

Statistical analysis was performed using GraphPad Prism software version 9.1. Categorical variables were analyzed using Fisher’s exact test, a nonparametric method for calculating exact probabilities (*p*-values) for small samples. A *p* value of <0.05 was considered statistically significant.

## 3. Results

To study the prevalence of influenza virus in 2020–2025, nasopharyngeal swabs from 2200 individuals of various ages (born 1935–2025) with various respiratory illnesses were analyzed. The proportion of women examined was 50.45% (1110 samples), the proportion of men examined was slightly less—49.55% (1090 samples).

**Figure 1:**
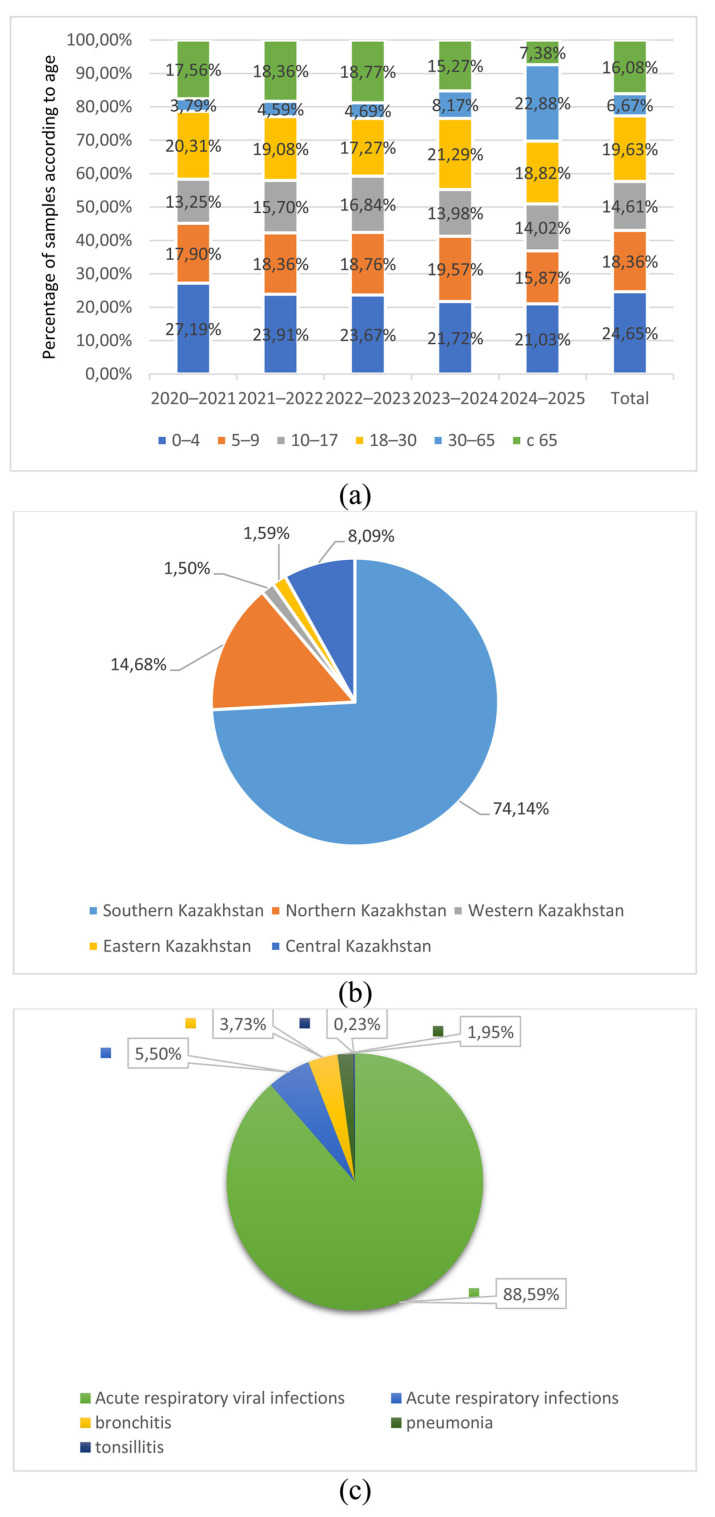
Distribution of samples according to age, sampling points, and primary diagnosis of the studied individuals. Distribution of samples according to (**a**) age, (**b**) sampling points, and (**c**) respiratory diseases of individuals studied.

The majority of samples were obtained from children aged 17 years and younger, accounting for 57.62% of the total sample. The second largest group consisted of individuals of working age (18–65 years)—26.30%. Individuals over 65 years of age accounted for approximately 16.08% (Figure 1a).

The majority of samples, 74.14% (n = 1631), were collected from healthcare facilities located in the southern part of Kazakhstan (Almaty, Zhambyl, Turkestan, and Kyzylorda regions), as this part of Kazakhstan has the largest population (Figure 1b). A smaller number of samples, 14.68% (n = 323), were collected from northern Kazakhstan, including Akmola, North Kazakhstan, Kostanay, and Pavlodar regions. The remaining regions of Kazakhstan collected the smallest number of samples, 11.18% (n = 246).

Depending on the diagnosis, the largest number of samples (nasopharyngeal swabs) were obtained from patients with acute respiratory viral infections and acute respiratory infections, accounting for 94.09%. Samples from patients diagnosed with bronchitis and pneumonia accounted for a smaller proportion (3.73% and 1.95%). Less than 1% of samples were obtained from patients with tonsillitis (0.23%) (Figure 1c).

Influenza virus genetic material in nasal swabs tested by RT-PCR was detected in 307 samples (13.95% of the total number of samples tested) (Table 1). IAVs were detected in 285 samples (12.95%), and influenza B virus in 22 samples (1.00%). Influenza A(H1N1)pdm09 virus RNA was detected in 79 samples (3.59%), and influenza A(H3N2) virus in 105 samples (4.77%). In 101 samples (4.59%) positive for IAVs, the subtype could not be determined. During the study period, A(H3N2) was the dominant causative agent among influenza viruses. However, during the 2021–2022 and 2023–2024 epidemic periods, its detection rate was highest, reaching 10.63% and 8.82%, respectively. Influenza A(H1N1)pdm09 was the primary etiologic agent among influenza viruses in 2023–2024; influenza A(H1N1)pdm09 RNA was detected in only one (0.22%) sample.

**Figure 2:**
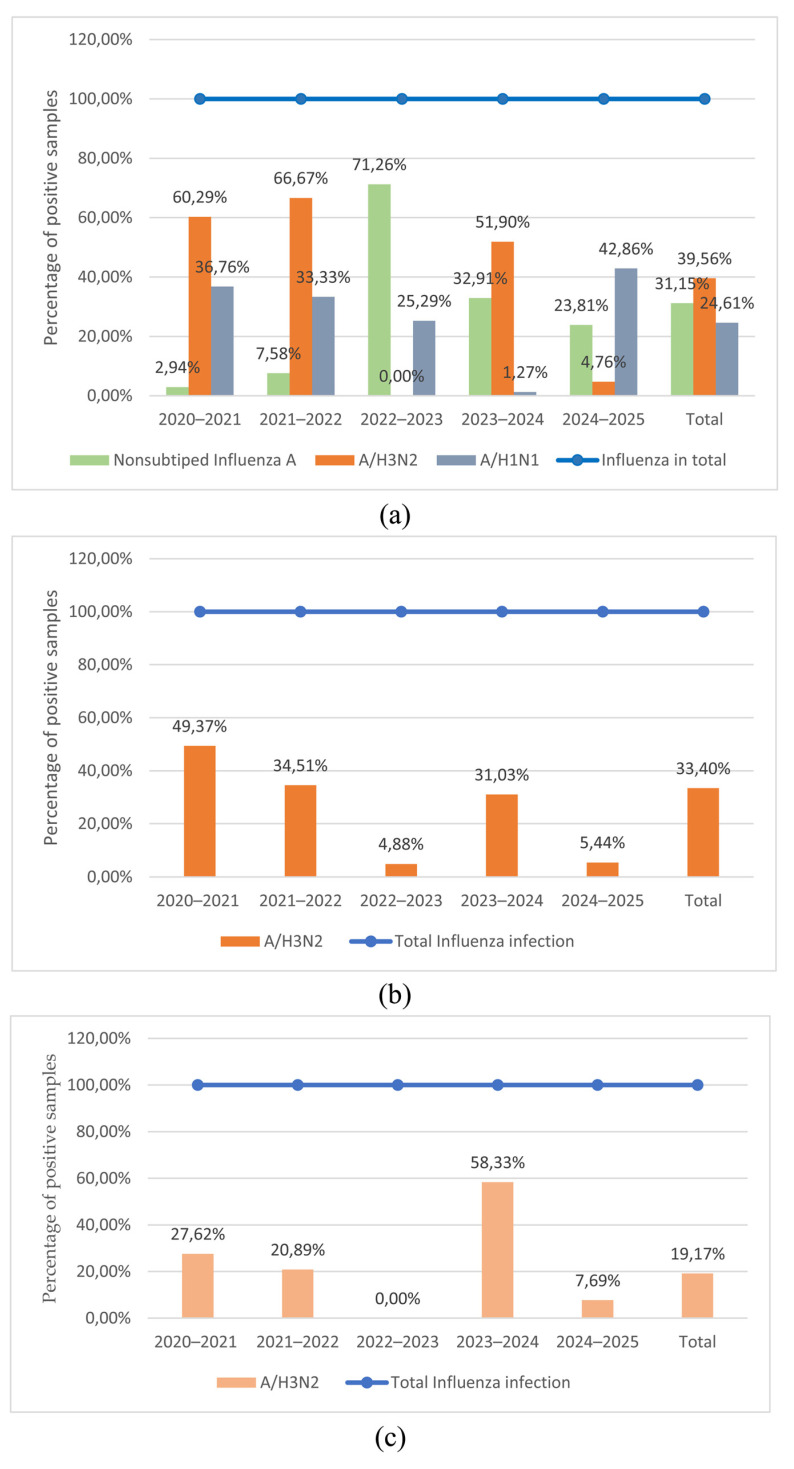
Determination of influenza A virus circulation in the 2020–2025 epidemic seasons. (**a**) detection of antigens of influenza A/H3N2 viruses using rtRT-PCR; (**b**) detection of antibodies against influenza viruses in blood serums using hemagglutination inhibition assay; (**c**) detection of antibodies against influenza viruses in blood serums using enzyme-linked immunosorbent assay.

The highest number of A(H3N2) viruses was detected in the epidemic years 2020–2021, 2021–2022, and 2023–2024; its detection rate from the number of positive samples per year was 60.29%, 66.67%, and 51.90%, respectively. In the structure of epidemic influenza incidence in 2023–2024, the IAV A(H3N2) was predominant; however, it was not detected in the 2022–2023 epidemic season (Figure 2a).

When analyzing the detection rate of influenza infection depending on age, it was found that the highest number of positive results 25.08% (n = 77) was detected in children under 4 years of age, while the lowest value 10.10% (n = 31) was found in elderly patients over 65 years of age (Table 2).

Among the blood serum samples, the largest number was obtained from adult patients. Individuals over 65 years of age constituted the largest group, accounting for 38.16% (n = 559) of the sera. The smallest number of samples were obtained from children aged 5–9 and 10–17 years: 2.05% (n = 30) and 3.38% (n = 48), respectively (Table 3).

A serological study of influenza circulation using HI revealed the presence of antihemagglutinins to the influenza A(H3N2) virus in 33.40% of blood sera (n = 313) out of the total number of influenza virus-positive samples (n = 937). The highest percentage of antibodies to viruses was detected in the 2020–2021, 2021–2022, and 2023–2024 epidemic seasons, amounting to 49.37%, 34.51%, and 31.03%, respectively (Figure 2b).

In the ELISA, antibodies to the influenza A(H3N2) virus were detected in 115 samples (19.17%) of the total number of samples positive for influenza virus antibodies (n = 600). The highest number of serum samples positive for the influenza A(H3N2) virus was detected during the 2023–2024 epidemic period, while no antibodies were detected in the serum during the previous 2022–2023 epidemic period (Figure 2c).

**Figure 3:**
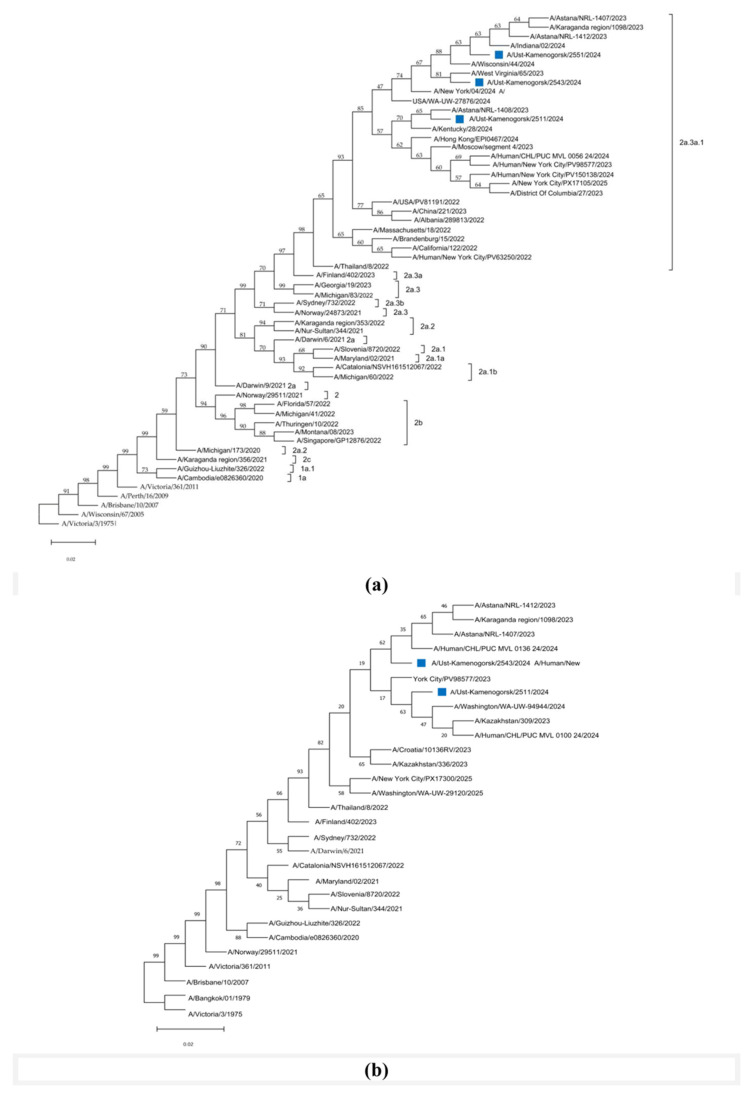
Molecular phylogenetic analysis for HA and NA. subclade classification by maximum likelihood method (**a**) the tree for HA with the highest log likelihood (−5190.81) is shown with using Hasegawa-Kishino-Yano (1985) model; (**b**) the tree for NA with the highest log likelihood (−3654.85) is shown. The blue colored squares represent the hemagglutinin nucleotide sequences of samples from Ust-Kamenogorsk (eastern part of Kazakhstan).

Direct sequencing of six rtRT-PCR-positive samples for the influenza A(H3N2) virus allowed us to obtain partial genome sequences of the IAVs in three samples. Complete genome assembly was performed for two samples, and HA and PB2 genes were assembled for one sample obtained from the eastern part of Kazakhstan, the city of Ust-Kamenogorsk. Molecular phylogenetic analysis for classification of the H3 subclade by the maximum likelihood method for three influenza viruses A/Ust-Kamenogorsk/2511/2024, A/Ust-Kamenogorsk/2543/2024, and A/Ust-Kamenogorsk/2551/2023 (H3N2) is shown in Figure 3.

The results of phylogenetic analysis showed that the HA and NA genes are more closely related to influenza viruses isolated in the United States (A/West Virginia/65/2023, A/Wisconsin/44/2024, A/New York/04/2024, A/USA/WA-UW-27876/2024, A/Kentucky/28/2024, A/Indiana/02/2024), China (A/Hong Kong/EPI0467/2024), the Russian Federation (A/Moscow/segment 4/2023), Kazakhstan (A/Astana/NRL-1408/2023, A/Astana/NRL-1412/2023, A/Astana/NRL-1407/2023 and A/Karaganda region/1098/2023), as well as with influenza virus strains circulating in different countries of the world, belonging to the 3C.2a1b.2a.2a.3a.1 clade. The strains were found to be closely related to A/District of Columbia/27/2023 and A/Croatia/10136RV/2023. These strains are recommended for use in vaccines for the 2025 influenza season. Like the A/Thailand/8/2022 and A/Massachusetts/18/2022 strains, they are considered reference viruses for the 2024 Southern Hemisphere and 2024–2025 influenza seasons in vaccines recommended by the WHO.

All analyzed A(H3N2) strains belonged to clade 2a.3a.1 and showed differences in 25–28 amino acid sequences of HA relative to the vaccine virus A/Darwin/6/2021. Moreover, the A/Ust-Kamenogorsk/2511/2024 virus had two unique substitutions (C1272T and A1654G), and A/Ust-Kamenogorsk/2551/2024–one (C1458T). In the NA gene sequence of the A/Ust-Kamenogorsk/2511/2024 and A/Ust-Kamenogorsk/2543/2024 viruses relative to the vaccine strain A/Darwin/6/2021, unique substitutions (A1160G and A643G) were also found.

The sequences were registered in the GISAID database under the accession numbers EPI_ISL_19186455, EPI_ISL_19186460, and EPI_ISL_19186463, respectively. Genotyping using the FluSurver analysis tools of the GISAID database revealed that the viruses have positions indicating their susceptibility to the NA inhibitors zanamivir, peramivir, oseltamivir, and laninamivir, as well as to the PA inhibitor baloxavir [25].

Thus, based on the results of RT-PCR testing of samples, circulation of influenza A (H1N1pdm09 and H3N2) and B viruses was established among the population in 2020–2025. The quantity of each virus varied depending on the epidemic phase. Influenza A (H3N2) virus was dominant during the epidemic periods of 2020–2021, 2021–2022, and 2023–2024. HI and ELISA data indicate circulation of influenza A and B viruses with a slight predominance of influenza A (H3N2). Based on the obtained sequencing data and phylogenetic analysis of influenza virus surface proteins, it was established that the strains circulating in Kazakhstan in 2023–2024 belong to clade 3C.2a1b.2a.2a.3a.1.

## 4. Discussion

Influenza surveillance shows that a single virus strain typically dominates during an epidemic [26]. Influenza viruses circulate worldwide and exhibit typical seasonal patterns. In the Northern and Southern Hemispheres, peak activity occurs during the winter months, while in tropical regions, influenza activity is more closely associated with the rainy season [27].

Up to 10% of children under 18 years of age and 4% of children under 65 years of age from the population seek outpatient care annually due to influenza-like illnesses [28].

The emergence of SARS-CoV-2 during the 2019–2020 COVID-19 pandemic led to a significant change in the circulation of other respiratory viruses, such as influenza viruses and respiratory syncytial virus, which temporarily ceased their circulation. The unprecedented decline in influenza virus circulation worldwide, including in Kazakhstan, during the COVID pandemic was apparently due to factors such as viral interference against the background of COVID-19 infection and the strengthening of anti-epidemic measures [29,30]. In the post-pandemic period, a partial change in the seasonality and epidemiology of respiratory viruses was observed, due to the influence of co-circulation of SARS-CoV-2 [31,32]. The 2020–2021 and 2021–2022 epidemic seasons saw changes in seasonal patterns, delayed peaks, and decreased genetic diversity of circulating A(H3N2) viruses. Following the lifting of SARS-CoV-2-related restrictions, the A(H3N2) virus caused an outbreak in southern China in the summer of 2022 and continues to circulate among humans [9]. A trend toward increased respiratory infection rates due to the easing of pandemic restrictions and changes in vaccination rates has also been observed in the United States [33].

In Kazakhstan, the A(H3N2) virus began to be detected starting from weeks 42 to 52 of 2021 [34]. In our study, the main group was formed by children from birth to adolescence (0–17 years), who made up 56.91% of the sample; the adult population (18–65) made up 26.86%, and the elderly ≥65 years old—16.23%. The high percentage of samples obtained in the pediatric age group may be associated with the number of children and adolescents who sought help from medical institutions, as well as their high share (about 33%) of the population of Kazakhstan at the beginning of 2025 [35]. The results of the present study correlate with the data obtained in previous studies [6,8,13,29,32].

**Figure 4:**
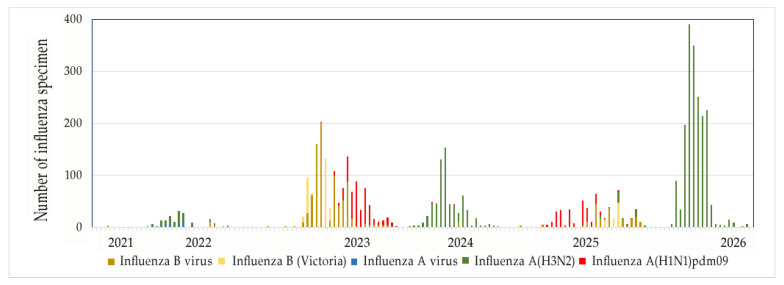
Influenza laboratory surveillance information. Data of World Health Organization for Kazakhstan. Virus detection by subtype reported to FluNet [36].

Our studies also revealed a trend toward seasonal patterns in influenza virus circulation. Thus, the proportion of A(H3N2) viruses increased during the 2020–2021, 2021–2022, and 2023–2024 epidemic periods, while only isolated cases were observed in 2022–2023 and 2024–2025. In serological studies, antibodies to influenza A(H3N2) viruses were also detected more frequently during the 2020–2021, 2021–2022, and 2023–2024 epidemic seasons. The obtained data correlate with the data presented in the information on laboratory epidemiological surveillance of influenza (Figure 4) [36]. When comparing our data with the WHO influenza surveillance data, a correlation is observed. However, the correlation for influenza A(H1N1) and B viruses is low. Significant correlation values were found only for H3N2 viruses. The majority of nasopharyngeal swab samples were obtained from children aged 17 years and younger, accounting for 57.62% of the total sample. The second largest group consisted of individuals of working age (18–64 years). Individuals over 65 years of age accounted for approximately 16.08% (Figure 1a). The highest number of positive samples (63.84%) for influenza viruses in RT-PCR was detected in children and adolescents; the lowest number of positive results for the influenza virus was found in older patients, which accounted for 10.10% of the total number of positive samples. This is consistent with data from other studies indicating the key role of school-age children in the seasonal spread of influenza due to their close contact in schools [37].

To confirm the data on virus spread monitoring and the presence of herd immunity in the population, serological studies were conducted using the HI and ELISA methods [38]. Serological analysis of 1465 blood serum samples revealed, in general, over the entire study period, a predominance of antibodies to IAVs (A/H1N1pdm09 and A/H3N2 in approximately equal amounts), while antibodies to influenza B virus were less common. The presence of antibodies in HI/ELISA to influenza A/H1N1pdm09 virus was detected in 26.04%/9.2% of samples, to influenza A/H3N2 virus in 29.59%/10.0% and to influenza B virus in 9.40%/9.6%. Antibodies to both IAVs (H1N1 + H3N2) were detected in 6.57%/3.8% of cases, and antibodies to both influenza A and influenza B viruses were detected simultaneously in 5.59%/9.7% of cases. In the period 2022–2023, antibodies to the influenza A/H3N2 virus were not detected in sera. Serological studies showed a low positive result for the presence of antihemagglutinins to A(H3N2) among children aged 0–17 years: 8.31% in the HI assay and 12.17% in the ELISA. Over time, a gradual increase in the detection of anti-influenza antibodies was observed. As has been shown during monitoring of SARS-CoV-2, multiple antigenic exposures to influenza viruses can lead to the development of reliable basic immunity. It is possible that after the formation of basic immunity by the age of 15, as a result of the accumulation of such effects, an increase in the coverage of humoral immunity occurs over time [37].

Serological studies play an important role in the diagnosis of infectious diseases, providing a rapid and accurate assessment of the immune status of patients [8,39]. However, in our investigations a direct comparison of influenza virus prevalence in nasopharyngeal swabs obtained by rtRT-PCR and the presence of antibodies detected by HI and ELISA methods is not possible, since nasopharyngeal and serological samples were obtained from different donors. Furthermore, vaccination cannot be ruled out as a factor contributing to seropositivity in serological tests, given the limited information on patients from whom samples were collected. Therefore, serological analysis data can be considered an indicator of influenza virus seropositivity in the population, rather than infection. This also applies to influenza type B, where a higher serological response was observed, which may indicate the effect of vaccination on immune development.

The prevalence of influenza A(H3N2) viruses in 2023–2024 was also detected in some other countries, such as Pakistan, Afghanistan, Uzbekistan, the Syrian Arab Republic, and Russia. In Kazakhstan, which has a long border with Russia, the predominant influenza virus pathogen during the 2023–2024 epidemic season was the A(H3N2) virus. Phylogenetic analysis of the HA gene sequences of influenza A(H3N2) viruses showed that they belong to one of the new genetic subclades of the 3C.2a1b.2a.2 clade [19,30].

All analyzed A(H3N2) strains belonged to clade 2a.3a.1 and showed differences in 25–28 amino acid sequences in the HA glycoprotein relative to the A/Darwin/6/2021 vaccine virus. Moreover, the A/Ust-Kamenogorsk/2511/2024 virus had two unique substitutions (C1272T and A1654G), and A/Ust-Kamenogorsk/2551/2024–one (C1458T). The studied viruses were found to have eight amino acid substitutions in HA1 (E50K, G53N, N96S, N122D, I140K, I192F, I223V, K276E), two in HA2 (N49S, M207V) and one in the signal peptide (T3A).

The identified substitutions in the HA2 subunit of the HA protein, at positions 49 and 207, and in the signal peptide at position 3 are frequently found in new variants of the influenza A(H3N2) virus and may affect its stability, ability to fuse with the cell, and the efficacy of vaccines developed for previous seasons. The N96S substitution led to the appearance of a potential N-glycosylation site, and the presence of the N122D mutation may indicate the loss of an N-glycosylation site [40]. The identified mutations in the influenza virus genome are relevant for monitoring in the 2025–2026 season.

Unique substitutions were also found in the NA gene sequence of A(H3N2): in IAV strains A/Ust-Kamenogorsk/2543/2024 (E83G, R150H, R400K) and A/Ust-Kamenogorsk/2511/2024 (R150H, D346N, N385D, R400K). Substitutions R150H and R400K are characteristic of many strains circulating in 2023–2025. In addition, nine potential N-glycosylation motifs were found in the NA sequence (61, 70, 86, 146, 200, 234, 245, 367, and 463), two of which (146 and 367) were located near the enzymatic active center [41]. However, no mutations indicating resistance to oseltamivir and zanamivir were detected in the NA gene of the A(H3N2) viruses studied.

The A/Ust-Kamenogorsk/2511/2024, A/Ust-Kamenogorsk/2543/2024, and A/Ust-Kamenogorsk/2551/2024 viruses differed from many viruses circulating in 2023/2024, including A/Croatia/10136RV/2023, in the positions of the HA1 amino acid substitutions 145 and 186. At these positions, they had the amino acids serine (S) and aspartic acid (D), corresponding to the A/Darwin/6/2021 virus, while A/Croatia/10136RV/2023 had the amino acids Asparagine (N) and Glycine (G), respectively, at these positions. At HA2 position 80, the A/Ust-Kamenogorsk/2543/2024 and A/Ust-Kamenogorsk/2551/2024 viruses had amino acid substitutions of asparagine (N) for isoleucine (I), while A/Ust-Kamenogorsk/2511/2024 and A/Croatia/10136RV/2023 had asparagine (N) for leucine (L) at this position, as well as in the vaccine virus A/Darwin/6/2021. In 207 of the HA2 sequence, the A/Ust-Kamenogorsk/2511/2024 virus had an amino acid substitution of methionine (M) for valine (V) at position 207 of the HA2 subunit of the hemagglutinin protein, unlike other viruses that had methionine (M) at this position.

In addition, mutations have also been detected in other genes of the A(H3N2) viruses. The following substitutions were identified: 5 substitutions in MP (M1:V219I, M2:P25L, M2:S31N, M2:L54F, M2:N82S) and NP (A131S, I136L, V197I, R236K, T472A), 12 substitutions in the NS1 sequence (E26N, L33I, A56S, V60A, E71G, V82A, M124I, S135N, D139G, I171V, K221E, K229E), three in PB1 (*758Q, F2:Q5L, F2:R21E), PB2 (PB2:S107N, PB2:I147T, PB2:V560I), seven in PA (N142K, R266K, I311M, Y321C, K605R, X:N142K, X:E209G). The identified mutation in the M2 gene S31N may indicate resistance to the drug rimantadine [42]. It should be noted that substitutions at positions 38, 34, and 28 of the PA protein associated with a decrease in baloxavir activity were not observed [40].

The three mutations detected in PB2 (PB2:S107N, PB2:I147T, PB2:V560I) are characteristic of IAVs (H3N2) of the K subclade (also known as J.2.4.1), which are dominant among the A(H3N2) representatives circulating in the 2025–2026 epidemic season in Europe and the USA.

It was noted that in previous epidemic seasons, H1N1pdm was dominant among circulating IAVs [43]. Whereas in the 2023–2024 epidemic season, A/H3N2 played a predominant role in the epidemic process. It was established that these strains are closely related to the IAV strains A/District of Columbia/27/2023 and A/Croatia/10136RV/2023 recommended for use as vaccines in 2025. In recent months, an increase in seasonal influenza activity has been observed worldwide, with influenza A(H3N2) viruses predominating. This increase coincides with the onset of winter in the Northern Hemisphere. Genetically modified influenza A(H3N2) viruses, known as subclade K viruses, have been detected in many countries [41].

Seasonal influenza, particularly A(H3N2) strains, significantly burdens healthcare systems worldwide, causing mild to severe illness, hospitalizations, and deaths. Although early surges in the Northern Hemisphere and increased H3N2 activity are typical seasonal phenomena, according to the WHO, seasonal influenza can place significant pressure on healthcare systems even in countries with temperate climates [41].

The prevalence of IAVs (H3N2) during the 2020–2025 acute respiratory viral infection epidemic seasons was assessed according to standardized international protocols. Unfortunately, our study has several limitations. First, the sample size is small relative to the population of Kazakhstan. Second, the number of samples collected across regions is uneven, which may influence the study results. The study used samples provided by healthcare workers by agreement. Sample collection was random and was conducted at healthcare facilities from different patients. No data was provided on whether blood swabs and serum samples were collected from the same patient. Age bias may also be present in the age distribution of the patients, although biological samples were collected in both pediatric institutions and primary health care centers. Most biological samples were obtained from large, densely populated cities located in southern Kazakhstan. Fewer samples were collected from other regions of the Republic of Kazakhstan. The frequency of antibody detection in serological studies to influenza B viruses was higher than the frequency of antigen detection in rtRT-PCR. This discrepancy may indicate both small sample sizes and the persistence of antibodies to the influenza virus after the previous epidemic or the presence of immunity after vaccination. However, it was established that the detection of influenza viruses A(H1N1)pdm, A(H3N2) and B confirms their circulation and key role in the epidemic process and indicates the need for continuous monitoring of the spread of respiratory viruses in various regions of Kazakhstan.

Due to the constantly changing nature of influenza viruses, WHO continues to emphasize the importance of year-round global epidemiological surveillance to detect and monitor virological, epidemiological, and clinical changes associated with the emergence or spread of influenza viruses that may impact human health, as well as the timely sharing of virus information for risk assessment. Countries are encouraged to remain vigilant regarding the threat of influenza viruses and monitor any unusual epidemiological trends [44].

## 5. Conclusions

A comprehensive study of respiratory virus circulation in Kazakhstan from 2020 to 2025 revealed the primary role of influenza A(H1N1)pdm09, A(H3N2), and B viruses in the epidemic process. Their ratios vary across epidemic seasons, with one strain predominating over another. It was established that the IAV strains of the 2023–2024 season (A/H3N2) belong to a specific genetic clade, J.2 or 3C.2a1b.2a.2a.3a.1. Continuous monitoring of such changes is critical to verifying the compatibility of circulating viruses with vaccine formulations. These studies directly impact the country’s biological security, enabling the selection of relevant strains for vaccine production and the optimization of national vaccination strategies.

Such research is essential for a timely response to virus evolution and the prevention of large-scale epidemics in the region.

## Figures and Tables

**Table 1:** Screening of nasopharyngeal samples collected in Kazakhstan in 2020–2025 using rtRT-PCR for Influenza viruses.

Virus	Number of PCR-Positive Virus Samples by Year					Total	*p*-Value
	2020–2021	2021–2022	2022–2023	2023–2024	2024–2025		
Number of tested samples	581	414	469	465	271	2200	0.056125 ^1^
Influenza A non-detected	2 (0.34) ^2^	5 (1.21)	62 (13.22)	26 (5.59)	5 (1.85)	101 (4.59)	0.095258
Influenza B	2 (0.34)	0(0.00)	3 (0.64)	11 (2.37)	6 (2.21)	22 (1.00)	0.078436
A(H1N1)pdm09	25 (4.30)	22 (5.31)	22 (4.69)	1 (0.22)	9 (3.32)	79 (3.59)	0.065263
A(H3N2)	41 (7.06)	44 (10.63)	0(0.00)	41 (8.82)	1 (0.37)	105 (4.77)	0.074824
Influenza in total	68 (11.70)	66 (15.94)	87 (18.55)	79 (16.99)	21 (7.75)	307 (13.95)	0.058481

^1^ *p*-values were tested by years for PCR-positive virus sample means with expected ones. ^2^ Data represents the number of positive samples (percentage).

**Table 2:** The number of rtRT-PCR-positive influenza virus samples collected in Kazakhstan in 2020–2025, broken down by age group.

Age Group (Years)	Number of PCR-Positive Samples for Influenza Virus					Influenza in Total
	Number of Tested Samples	Non- Subtyped Influenza A	Influenza B	A(H1N1) pdm09	A(H3N2)	
0–4	526 (23.91) ^1^	25 (24.75)	6 (27.27)	20 (25.32)	26 (24.76)	77 (25.08)
5–9	402 (18.27)	22 (21.78)	5 (22.73)	17 (21.52)	23 (21.90)	67 (21.82)
10–17	324 (14.73)	17 (16.83)	4 (18.18)	13 (16.46)	18 (17.14)	52 (16.94)
18–30	428 (19.45)	13 (12.87)	3 (13.64)	10 (12.66)	14 (13.33)	40 (13.03)
30–65	163 (7.41)	13 (12.87)	3 (13.64)	11 (13.92)	13 (12.38)	40 (13.03)
≥65	357 (16.23)	11 (10.89)	1 (4.55)	8 (10.13)	11 (10.48)	31 (10.10)
Total	2200	101	22	79	105	307
*p*- value	0.055533	0.055552	0.057957	0.055714	0.055658	0.055748

^1^ Data represents the number of positive samples (percentage).

**Table 3:** Detection of antibodies against influenza virus strains in different age groups using HI assay and ELISA during the 2020–2025 epidemic periods.

Age Group (Years)	Sample Size	Numbers of Participants That Are Immune to Influenza Viruses					
		Other Influenza A Virus		A(H3N2)		Total	
		HI	ELISA	HI	ELISA	HI	ELISA
0–4	59 (100.0) ^1^	12 (20.34%)	7 (11.86)	12 (27.12)	5 (8.47)	22 (37.29)	12 (20.34)
5–9	30 (100.0)	5 (16.67)	2 (6.67)	2 (6.67)	2 (6.67)	9 (30.00)	4 (13.33)
10–17	48 (100.0)	18 (37.50)	10 (20.83)	6 (12.50)	7 (14.58)	30 62.50)	17 (35.42)
18–30	296 (100.0)	71 (23.99)	43 (14.53)	25 (8.45)	16 (5.41)	113 (38.18)	59 (19.93)
30–65	473 (100.0)	201 (42.49)	139 (29.39)	158 (33.40)	50 (10.57)	345 (72.94)	189 (39.96)
≥65	559 (100.0)	170 (30.41)	126 (22.54)	106 (18.96)	35 (6.26)	271 (48.48)	161 (28.80)
Total	1465 (100.0)	477 (32.56)	327 (22.32)	313 (21.37)	115 (7.85)	790 (53.92)	442 (30.17)
*p*- value		0.352496		0.033752		0.142224	

HI: hemagglutination inhibition assay; ELISA: enzyme-linked immunosorbent assay. ^1^ Data represent the number of positive samples (percentage).

## Data Availability

The original contributions presented in this study are included in the article. Further inquiries can be directed to the corresponding author.

## Abbreviations

The following abbreviations are used in this manuscript:

CDC	Centers for Disease Control and Prevention
WHO	World Health Organization
IAV	Influenza A virus
rtRT-PCR	Real-time polymerase chain reaction
ELISA	Enzyme-linked immunosorbent assay
HI	Hemagglutinating activity inhibition
